# Novel Methods for Recording Stress-Strain Curves in Proton Irradiated Material

**DOI:** 10.1038/s41598-020-62241-2

**Published:** 2020-03-24

**Authors:** Albert D. Smith, Jack M. Donoghue, Alistair J. W. Garner, David Lunt, Allan Harte, Keith Wilford, Philip J. Withers, Michael Preuss

**Affiliations:** 1 School of Materials, The University of Manchester, Manchester, M13 9PL UK; 2 United Kingdom Atomic Energy Authority Culham Science Centre, Abingdon, OX14 3DB UK; 3 Rolls-Royce plc, Raynesway, Derby, DE21 7WA UK; 4 Henry Royce Institute, School of Materials, The University of Manchester, Manchester, M13 9PL UK

**Keywords:** Mechanical properties, Metals and alloys, Nuclear energy, Mechanical engineering, Characterization and analytical techniques, Scanning electron microscopy

## Abstract

Proton irradiation is often used as a proxy for neutron irradiation but the irradiated layer is typically <50 μm deep; this presents a problem when trying to obtain mechanical test data as a function of irradiation level. Two novel methodologies have been developed to record stress-strain curves for thin proton-irradiated surface layers of SA-508-4N ferritic steel. In the first case, *in-situ* loading experiments are carried out using a combination of X-ray diffraction and digital image correlation on the near surface region in order to measure stress and strain, thereby eliminating the influence of the non-irradiated volume. The second approach is to manufacture small-scale tensile specimens containing only the proton irradiated volume but approaching the smallest representative volume of the material. This is achieved by high-speed focused ion beam (FIB) milling though the application of a Xe^+^ Plasma-FIB (PFIB). It is demonstrated that both techniques are capable of recording the early stage of uniaxial flow behaviour of the irradiated material with sufficient accuracy providing a measure of irradiation-induced shift of yield strength, strain hardening and tensile strength.

## Introduction

In order to increase the power capacity and operational life of modern reactor designs, a better understanding of the performance of materials under high levels of irradiation damage is required. Displacement damage leads to degradation of mechanical properties through irradiation-induced hardening and reduction in strain hardening and strain to failure^1,2^. The most reliable method of testing materials behaviour under reactor conditions is achieved by placing surveillance specimens within an operating or test reactor, which are removed and tested periodically^3^. However, the high cost and long duration of these tests can be prohibitive^4^. The low displacement efficiency of neutrons leads to experiments running from months to years in order to reach relevant levels of damage. In addition, high post-irradiation activity can require long cooling-off periods, or expensive radiation handling protocols, to safely perform off-site examination, increasing test duration further^5^. This leads to a long turnaround time for specimens and can be prohibitive to studies requiring a wide range of irradiating conditions or new alloy designs. As a result, more cost effective, alternative forms of irradiation, particularly proton and heavy ion irradiation, have been developed in order to mimic the effects of neutron irradiation over shorter timescales with reduced activation.

Protons produced by a spallation source typically have energies in the order of 100 s of MeV, inducing displacement damage similar to the effects of neutrons. Such high kinetic energy has the advantage of through-thickness irradiation of bulk specimens, so can be analysed using standard mechanical testing methods^6–12^. However, this approach suffers from the same limitations as neutron irradiation, in terms of poor displacement efficiency and high residual post irradiation activity. This drives up experimental expense and limits the availability of equipment. Lower energy proton beams (e.g. generated by Pelletron) induce similar displacement damage effects with a reduced post-irradiation activity, allowing for easy handling, transportation and testing^13,14^. The reduced energy of the incident beam is more efficient at generating displacement damage and so displacement damage rates can be increased by up to an order of magnitude. Depending on the energy, low energy protons can attain damage levels in a matter of hours that would take months to achieve in a test reactor or on a spallation beamline. However, the benefits of low energy proton irradiation comes at the expense of significantly reduced penetration depth^15^.

The limited penetration depth of proton irradiation makes measurement of mechanical properties difficult using established techniques^16,17^. Over the last decade, the use of Ga^+^ focussed ion beam (FIB) instruments has allowed for the preparation of small scale samples from proton irradiated layers^18–23^. The mechanical properties of these samples are then tested using a MEMS chip or piezo-actuated test rig without contributions from the non-irradiated volume. This development has allowed studies to take advantage of the increased dose rates and to probe changes in properties previously only attainable using indentation testing^24–28^. However, milling rates are slow because for Ga^+^ FIBs the useable milling currents are limited due to the point source of Ga^+^ ions. Consequently, specimen diameters achievable in a practical time frame are limited to ~10 µm. For most engineering materials, the maximum achievable scale is therefore in the order of single to only a few grains. Hence, the technique is most applicable to single and bi-crystal investigations rather than representing the bulk response of polycrystalline specimens. It has been demonstrated that small scale specimens exhibit an increased hardening inversely proportional to specimen diameter^29–33^. In order to obtain a bulk response, specimens require a minimum length scale sufficient to overcome size reduction effects. The optimum specimen size would have the smallest representative volume (SRV), which would retain the benefits of scale reduction whilst exhibiting bulk behaviour^34^.

Recent work by the authors has explored two techniques to increase the sampling volume in low energy proton irradiated samples prepared for mechanical testing^35,36^. The first, an adaptation of the technique outlined by Foecke *et al*.^37^, combines *in-situ* X-ray diffraction stress measurement and digital image correlation (DIC) to construct uniaxial flow curves^35^. Laboratory-based sin^2^Ѱ diffraction stress measurement relies on the limited X-ray penetration depth, which for steels is of the same order as the proton irradiated layer (~35 μm at 3 MeV)^35^. Calibration of the lattice response to applied stress, using the methodology outlined in ASTM E1426 -98^38^, allows for the calculation of stresses in the same material under the assumption that the lattice response to applied stress is consistent between tests. Strain is calculated using DIC on optical images collected in the gauge area in parallel to the stress measurements. DIC allows for non-contact measurement of the in-plane (total) strain on the surface and therefore both stress and strain data are collected from the near surface region that is affected by irradiation hardening. The second method exploits the significantly faster milling rates that can be achieved for plasma Xe^+^ focussed ion beam (PFIB) technology compared to Ga^+^ FIBs due to the higher milling currents^39^. This enables large scale machining of material at milling rates some 100 times that of Ga^+^ FIB^40,41^. A methodology has been developed for the manufacture of samples with a gauge cross sections in the 90 µm^2^ range^36^. It has been demonstrated that such specimens exhibit a proof stress comparable to a standardised bulk test, however, they did display a size-dependent plastic deformation response^36^. A methodology has been developed for the manufacture of samples with dimensions approaching the SRV, with a gauge section in the meso-scale exhibiting bulk behaviour in all aspects except strain hardening^36^. The present study aims to apply and compare side by side these novel techniques for the first time in order to record the mechanical response of 3 MeV proton irradiated SA508-4N steel (a candidate RPV steel), irradiated to a depth of approximately 30 μm.

## Results

**Figure 1:**
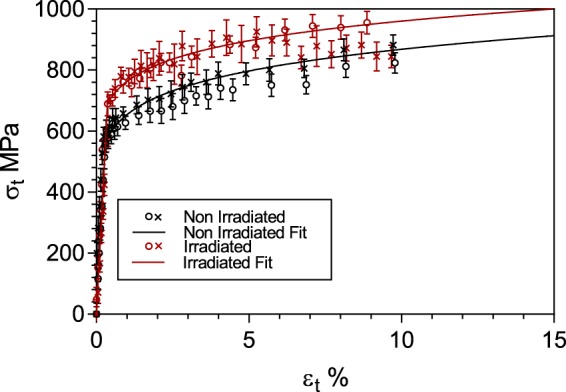
True stress – true strain curves generated by XRD/ DIC technique for irradiated and non-irradiated specimens, error bars are gradient error in sin^2^Ѱ vs. d-spacing plot. Red markers are irradiated, black markers are non-irradiated collected in ref. ^35^, with crosses and open circles representing different specimens. Solid lines depict fitted curves assuming power-law hardening from each technique for irradiated and non-irradiated states.

A comparison between the flow curves generated from the XRD/DIC technique in the irradiated region, irradiated using 3 MeV protons to the range of  around 10 mdpa according to SRIM calculations at 330 ± 3 °C and a flux of 2.3 × 10^14^ cm^−2^ s^−1^, and from the non-irradiated material from ref. ^36^ is shown in Fig. 1. The proof stresses of the irradiated samples were obtained using the standard 0.2% offset, providing an average value of 770 ± 29 MPa, whereas the proof stress of the non-irradiated material was 633 ± 22 MPa. This value agrees well with the estimated increase in yield stress of 110 MPa inferred from indentation testing, which was calculated using the relationship from ref. ^25^:

1 $$\Delta {\sigma }_{y}=3.03\Delta {H}_{V}$$

where, $\Delta {\sigma }_{y}$ is the calculated increase in yield stress; $\Delta {H}_{V}$ is the measured increase in Vickers hardness due to irradiation damage and 3.03 is the correlation function for ferritic steel^25^.

Strain hardening was calculated for the irradiated specimens using the log-log gradient of the plastic regime between proof and peak stress. The stress-strain curves exhibited a slight reduction of ~0.013 in strain hardening, relative to the non-irradiated material (~11% change). Tensile strength, taken as the peak stress, remained unchanged at ~875 MPa, while the strength coefficient (K) was observed to decrease by 89 MPa from 1120 MPa. Fitted curves generated using Hooke’s law and a power law hardening model are shown in Fig. 1, with plastic behaviour characterised by the Hollomon equation:

2 $${\sigma }_{t}=K{\varepsilon }^{n}$$

where ${\sigma }_{t}$ is true stress, K is the strength coefficient and *n* is the strain hardening exponent.

**Figure 2:**
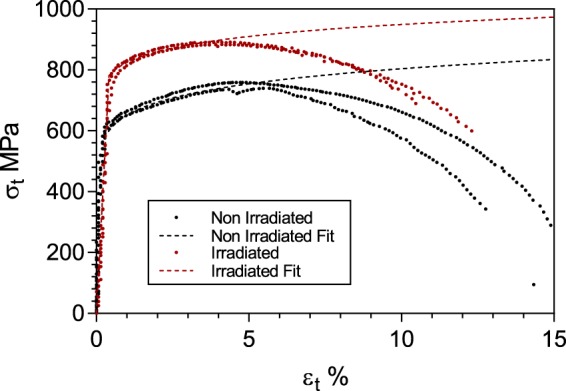
Stress – strain plots of *in-situ* testing of small-scale specimens prepared by PFIB. Red markers are irradiated and black markers are non-irradiated collected in ref. ^36^. Dashed lines depict fitted curves assuming power-law hardening from each technique for irradiated and non-irradiated states. True stress and true strain are only valid during uniform plastic deformation, so can be considered as engineering stress and strain beyond the plastic instability strain.

Figure 2 illustrates the flow curves recorded from the micro-tensile specimens prepared from the proton irradiated region using the Xe^+^ PFIB method described above. Here the proof stress was recorded as 807 ± 9 MPa, exhibiting an irradiation hardening of 161 ± 9 MPa. This is approximately 24 MPa higher than the average value of hardening reported using the XRD and DIC technique, and ~50 MPa more than that inferred from indentation testing. The strain hardening exponent was calculated to be 0.06, a reduction of 0.034 relative to the non-irradiated state. It must be emphasised that the strain hardening exponent recorded in the non-irradiated specimen is already lower for the micro-tensile specimen than in the bulk samples^36^. The exponent is approximately 21% lower than that recorded using the XRD-DIC method and the standardised method^35^. Tensile strength was measured to be to 866 ± 3 MPa using this technique, corresponding to an increase relative to the non-irradiated state of 135 MPa; furthermore, the strength coefficient was increased to 1093 MPa from 992 MPa.

**Figure 3:**
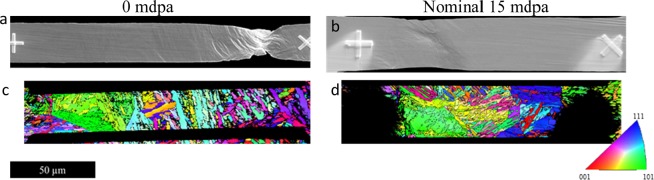
Post necking behaviour of non-irradiated and irradiated micro-tensile specimens. (**a**,**b**) Secondary electron images taken prior to failure; (**c**,**d**) Electron backscattered diffraction orientation maps collected prior to testing of non-irradiated and irradiated specimens (displayed in the inverse pole figure colour scheme) projects plane normal parallel to the loading direction; a & c are adapted from^36^.

**Figure 4:**
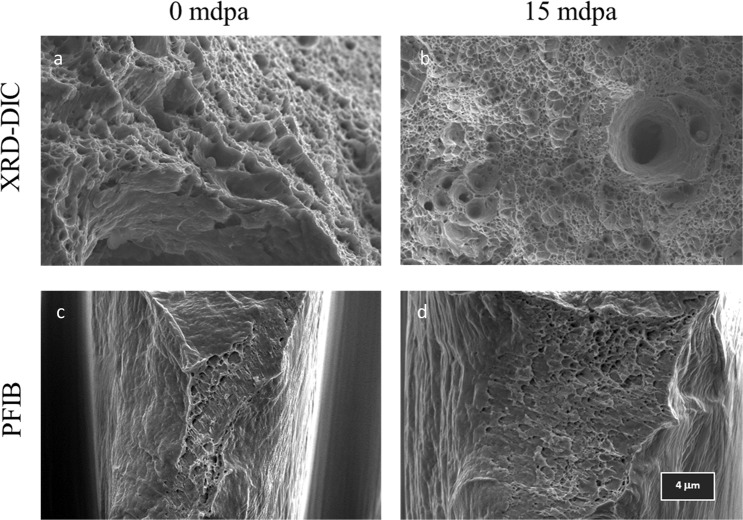
Secondary electron images of fracture surfaces for each sample (**a**) non-irradiated XRD/DIC; (**b**) irradiated XRD/DIC; (**c**) micro-tensile (PFIB) non-irradiated (**d**) micro-tensile (PFIB) irradiated.

SEM images (Fig. 3a,b) of each PFIB specimen prior to failure show some apparent differences between the non-irradiated and irradiated specimen, Fig. 3c,d show corresponding orientation maps for each of the images shown. The irradiated specimen displays planar slip in the region of the neck which is indicative of dislocation channelling due to defect clearing during deformation, while the non-irradiated shows more diffuse homogeneous deformation^42^. Fracture surfaces for irradiated and non-irradiated specimens, tested using both techniques, are displayed in Fig. 4. The region displayed for the irradiated sample, tested using the combination of XRD and DIC, is the flat portion of the dose profile. All examples illustrate that both non-irradiated and irradiated samples failed in the same way, by ductile void coalescence. However, fractography of the micro-tensile samples highlights the triaxial stress state at the neck during failure. This is amplified due to the removal of constraint, resulting in drawn out elongated cavitation, which extends in the direction of shearing. It is also notable that the reduction in area is significantly larger in the non-irradiated sample than the irradiated, corresponding to 95% and 71% respectively.

## Discussion

Both techniques have demonstrated that they are capable of recording a change in mechanical properties induced by displacement damage from proton irradiation. An increase in yield stress and decrease in strain hardening was documented using both XRD-DIC and micro-tensile testing.

**Figure 5:**
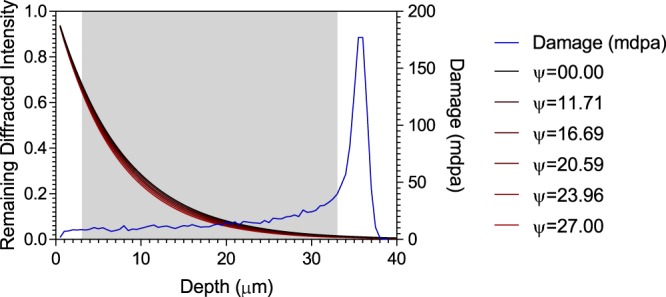
Technique dependent sampling depth relative to the SRIM damage profile for both techniques; shaded region corresponds to sampling range of PFIB micro-tensile specimens.

Due to the variance in relative sampling volumes for each technique, the recorded values of yield shift are slightly different despite being irradiated under the same conditions. This difference corresponds to a variation of nearly 40 MPa, with the specimens prepared using PFIB having the largest yield shift. Although the samples prepared using PFIB accumulate some surface damage due to ion beam milling, previous observations have shown that the damage layer is approximately 40% shallower compared to that induced using Ga^+^ FIB, being in the order of 10 s of nano meters^43,44^. This represents approximately 5 × 10^−3^% of the sample volume which is not thought to contribute significantly to the measured shift. Preparation of the specimens will also introduce heat, temperature increases of 100 °C are predicted at 20 nA μm^−1^ for Ga^+^ FIB milling of materials with a similar thermal conductivity to the alloy in the present study, with the range of the heated zone calculated as approximately twice the diameter of the beam spot^45^. In addition, post irradiation annealing studies of ferritic steel alloys have shown that below 300 °C there is little defect annihilation from increased thermal mobility^46^. While it is likely that high current milling with Xe^−^ ions lead to higher temperatures, the annealing footprint would be so localised that it is not expected to have an effect on the mechanical properties recorded. The relatively small differences between proof stresses in the non-irradiated dataset indicates that the variation evident in the irradiated sample is not a characteristic intrinsic to the method of preparation. This variation in yield stress is considered to be due to the difference in relative sampling volumes between the techniques, each effectively measuring a different dose. Figure 5 illustrates this; the area shaded in grey highlights the region sampled in the micro-tensile specimens and the plots shaded red through to black illustrate the attenuation of X-rays at each Ѱ-tilt. The penetration depth as a function of tilt was calculated using^47^:

3 $${G}_{x}=1-exp\left\{-\mu x\left[\frac{1}{sin(\theta +\psi )}+\frac{1}{sin(\theta -\psi )}\right]\right\}$$

where ${G}_{x}$ is the total diffracted intensity at a depth of $x$; $\mu$ is the linear absorption coefficient; $\theta$ is the diffraction half angle and $\psi$ is the tilt angle relative to the sample surface.

In contrast, the sampling volume of the micro-tensile specimens were sampled from the region ranging from 3–33 µm, with uniform sampling over this segment of the dose profile. Weighted dose (*D*_*w*_) was calculated by integration to take account of both the non-linear sampling of XRD and the non-linear dose profile simulated using SRIM:

4 $${D}_{w}=\frac{{\sum }^{}{D}_{i}{G}_{i}}{{\sum }^{}{G}_{i}}$$

and

5 $${D}_{w}=\frac{{\sum }^{}{D}_{i}}{N}$$

where *D*_*i*_ and *G*_*i*_ are dose and diffracted intensity diffracted intensity respectively at each bin and *N* is the number of bins. Bins were set at 500 nm increments, with *D* and *G* calculated as a trapezium to increase accuracy.

Each dose, weighted for Ѱ-tilts, gave an average of 9.9 mdpa calculated for those samples measured using XRD. Dose was found to be inversely proportional to Ѱ-tilt angle due to the larger penetration depth at lower angles and ranged by 0.5 mdpa from 10.2 mdpa at Ѱ = 0° to 9.7 mdpa at Ѱ = 27°. The range of doses were relatively narrow due to the exclusion of the stopping peak. Samples prepared using PFIB included some of the base of calculated damage peak, this gave a weighted dose of 15.6 mdpa. Given the weighted dose is 37% larger and the yield shift measured was greater in the specimens prepared by PFIB, the difference in proof stress is most likely due to the sampling volume.

Both techniques revealed a measured decrease in strain hardening due to irradiation damage. This behaviour is consistent with previous observations that show that irradiation damage has a deleterious effect as a function of dose^48^ up to the critical value where strain hardening drops to zero and necking occurs at yield^49^. There is a distinct difference between the measured values using both approaches, however this is not thought to be due to differences in the sampling volumes as with the yield shift.

The strain hardening exponent measured from stress strain curves recorded by XRD-DIC in the non-irradiated condition is comparable to that for the bulk^35^. However, when considering the differences in yield points and hardening rates of the non-irradiated “substrate” and irradiated layer, this may lead to a deviation from the results of a sample that was irradiated through thickness. The lattice response of dual-phase or composite materials during *in-situ* loading is analogous to the current study. Tensile deformation of these dual property materials will exhibit a linear response to applied stress up to the yield point of the softest constituent. Beyond which, load is understood to partition and will be transferred onto the harder of the constituents^50–53^. Hence, this will result in a larger stress recorded in the harder constituent of a dual property material than would be the case at the same level of strain if the same constituent were isolated. This has implications on the validity of the measured strain hardening in the irradiated layer. Due to partitioning, each increment of strain would result in a larger level of stress, which in turn will affect the calculated strain hardening parameter and is further complicated when considering the property gradients present in proton-irradiated material (Fig. 5). A good indicator of the presence of load partitioning is the linearity of the elastic range for the irradiated layer, which would result in an inflection upon the yielding of the non-irradiated substrate^53^. Although this inflection was not observed, it is difficult to state whether or not partitioning is occurring with any certainty, as there is only one measurement point between the yield stress and proportional limits of each layer. It may well be the case that the effect of partitioning is diminished due to the low volume fraction of irradiated material (~4%). An *in-situ* study of dual phase 737-DP and 775-DP steels has reported the effect of load partitioning on strain hardening was smaller in the alloy with a lower volume fraction of the harder martensite phase^54^.

The strain hardening parameter of non-irradiated small-scale specimens, prepared using PFIB, has already been demonstrated to be far lower than that of bulk tests^36^. This is thought to be due to the high ratio of grains intersecting the surface to those fully constrained in bulk. Although the relative volumes of the irradiated specimens are approximately 50% larger than the non-irradiated, the smallest dimension is the same in both sets (thickness). Under the assumption that martensite block boundaries control the hardening parameter^55,56^, the ratio of smallest specimen thickness to “effective grain” diameter (*t/d*) remains the same for both non-irradiated and irradiated samples. Hence, despite the larger gauge volume of the irradiated samples, they are expected to be subject to the same scale dependant diminished hardening as was observed in the non-irradiated set^36^. The low t/d ratio is thought to be the origin of the diminished hardening capacity, with an increased number of grains intersecting the sample surface an easy route for dislocation annihilation is provided which moderates the rate of accumulated backstress^57^. Whereas, specimens with a higher *t/d* ratio (i.e. bulk) will harden at a higher rate due to the contributions of back stresses generated by dislocations accumulating in the larger fraction of fully constrained grains^19,58^. It has been shown that the strain hardening parameter (n) raises with increased *t/d* before stabilising and accurately reflecting bulk hardening^59^, the position and shape of this threshold value can vary considerably between materials^60^.

Work by Byun and Farrell illustrates that irradiated materials exhibit a UTS that is typically similar to that of the non-irradiated material^10,49,61–63^. As dose is increased, the yield stress increases and is coupled with a reduction of strain hardening, at the critical point satisfying σ_y_ ≥ σ_u_ a specimen will experience prompt necking at yield. Whilst the yield shifts in the current work were insufficient to exceed this threshold value, UTS measured in specimens using both approaches were close to the one of bulk non-irradiated in all but the non-irradiated samples prepared using PFIB. Specimens prepared by top-down PFIB milling invariably exhibit side-wall tapering due to Xe^+^ ion beam profile and material redeposition. UTS has been shown to be inversely proportional to gauge section taper in non-irradiated samples tested in tension^36^. This implies that taper may have been significantly lower in the micro-tensile irradiated specimens, however, they were prepared using the same methodology as in^36^. It may also be the case that stress has been raised above the threshold regardless of the geometric effect of taper, providing an accurate UTS.

The presented techniques are subject to intrinsic and extrinsic sources of error, some of which have already been discussed, including geometry, scale and load partitioning. Misalignment of samples in the 5 kN loading rig used in the XRD-DIC experiments, as with all gripped sample loading rigs, can introduce scatter in recorded properties. This scatter is due to a strain gradient placing the sample in shear as a result of off-axis loading^64^. At the larger scale, this can be minimised by the use of guide pins, specialised grips or calibration, outlined in ASTME1012-05^65^. However, at the sub-millimetre length scale, specimen alignment becomes a significant issue^34,36,66^. In order to ensure accurate alignment with the loading axis, specimens were prepared within the fixture that was directly attached to the tensile testing apparatus and milling was performed with care to ensure the samples remain parallel with the external fixture. Even with a well aligned gauge length, off-axis loading may result from a mismatched pair of loading contact surfaces. In a compression test this would correspond to the punch and the top of the specimen and in tension would be the loading pin and loop interior. Due to the aforementioned sidewall tapering arising from FIB milling, mismatched surfaces are inevitable without employing overtilt during preparation^34^.

The damage profile that is intrinsic to proton irradiated materials is also likely to introduce uncertainty in the measured properties. Each approach samples a non-linear dose which can be considered as a property gradient over the sampling range. As the softer regions near the proton-beam entry surface yield, localised deformation will occur and, even in a perfectly aligned configuration, the test will no longer be truly uniaxial. In order to avoid this localisation, the use of a significantly smaller specimen thickness than the irradiation stopping range has been suggested^67^, however, a smaller specimen diameter would lead to a less representative sampling of grains. This could be improved by increasing the energy of the proton beam but the advantages of an improved penetration depth must be weighed against the disadvantages of an increased activation cross section and dramatic reduction in irradiation rate. Although the sampling volume of XRD stress measurement has been shown to be weighted to the flatter region of the dose profile in this study, property gradients within the measured volume may also contribute to the error in the measurement. Non-zero out of plane shear stresses can introduce a phenomena referred to as psi-splitting, which is a breakdown in the linearity of the sin^2^ ψ plot^47^. The split manifests as an upwards and downwards deviation from the centre line at positive and negative tilts, this will increase errors in the gradient used to calculate stress. This was not observed in the present work; however, it may become apparent in specimens that possess a steeper gradient. Therefore, it may not be suitable to apply the technique to irradiation techniques yielding even a thinner irradiated layer such as heavy ion irradiation.

## Conclusions

Two novel methods for testing the mechanical properties in polycrystalline samples of proton irradiated material (and potentially other surface modifications) have been applied for the first time to measure changes in yield and strain hardening behaviour and the invariance of UTS. The main conclusions are as follows:

- Both techniques have been used successfully to record stress-strain curves of proton irradiated material but significant differences in those curves were detected between the two methodologies. It is clear that additional experimentation is required to further calibrate both techniques, the most important of which are: an investigation into load partitioning between non-irradiated/irradiated layers and a systematic assessment of the smallest volume required to represent bulk behaviour.
- The combination of XRD and DIC for *in-situ* measurement of stress and strain provides a relatively low-cost method of recording flow curves in proton-irradiated material. The advantage to utilising this technique is that the results are comparable to those collected using standardised testing. Further sensitivity studies are necessary to identify the potential influence of the soft substrate on the yield behaviour of the irradiated layer.
- The use of Xe^+^ PFIB has been shown to be an effective method for the manufacture of mesoscale proton irradiated specimens. However, the achievable gauge volume from a tensile sample machined in this way is still very small making it most applicable to materials with grains around the 1 micron range. In the present case, SA508-4N martensitic-bainitic steel displayed a sub-micron morphology and despite such small structure the flow curves indicated a lack of constraint in the gauge volume. This is understood to be due to an insufficient number of effective dislocation barriers within the volume to facilitate the accumulation of bulk representative back stresses.

In summary, both techniques provide a method of obtaining tensile stress-strain data from the proton irradiated layer in a sampling volume that was previously impossible using preceding techniques offering complementary insights into the effect of the dose distribution on mechanical properties.

## Methods

### Material and specimen preparation

The material in this investigation was SA508-4N martensitic-bainitic steel, supplied by Rolls-Royce plc. Dog bone tensile samples were prepared with a 27 mm × 2 mm ×1 mm gauge section by electrical discharge machining (EDM). Coupons for the preparation of specimens by PFIB were also prepared by EDM to the dimensions 27 mm ×3 mm ×1 mm. All specimens were ground to remove the recast layer and subsequently polished using a standard metallurgical preparation route.

### Irradiation experiment

**Figure 6:**
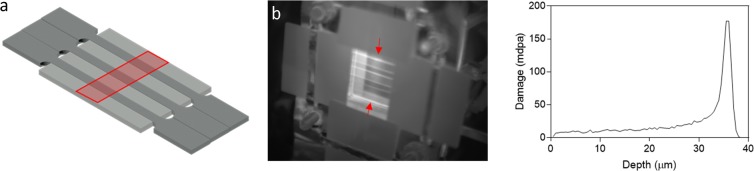
(**a**) Schematic showing the proton irradiation set up of the samples with a gauge section of 27 × 2 × 1 mm^3 ^^74^, (**b**) thermal image of specimens mounted on the end station during irradiation, the arrows indicate the beam position and (**c**) SRIM simulation for current collected on stage during proton irradiation to achieve nominal damage level of 10 mdpa.

**Figure 7:**
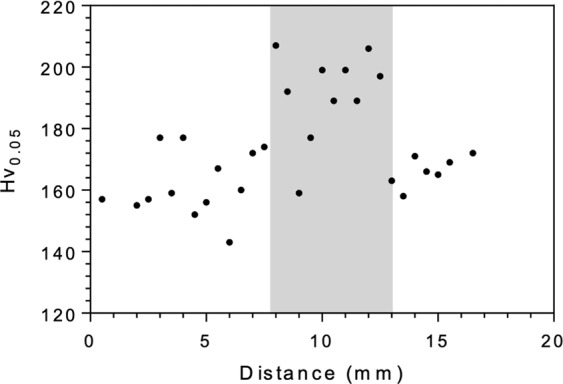
Typical indentation profile of irradiated region taken at 0.05 H_v_ (~18.42 µm diagonal), with an average irradiation hardening of 36 H_v._; shaded area denotes the irradiated region.

The irradiation experiments were carried out using the DAFNE 5MV Tandem Pelletron at the University of Manchester Dalton Cumbria Facility^68,69^. The specimen setup during irradiation is illustrated in Fig. 6(a,b). A “jigsaw” configuration was designed to reduce leakage of the liquid indium eutectic behind the sample used as a heat sink during irradiation. Proton irradiation was carried out at 3 MeV at a flux of 2.3 × 10^14^ cm^−2^s^−1^ to obtain a fluence of 8.43 × 10^17^ cm^−2^, with the beam rastered over a 5 × 25 mm^2^ area. The beam was over-scanned by 40% onto the aperture veins to produce hard edges in the irradiated region. Proton stopping range calculations were performed using SRIM^70^ with the ‘quick Kinchin–Pease calculation’^71^. The calculation predicted a stopping peak at ~36 µm, with the damage at 60% of the penetration depth of the stopping peak taken as the nominal damage (Fig. 6c). The temperature was monitored using a pyrometer, which was pre-calibrated according to the method outlined by Wady *et al*.^68^. The mean temperature was recorded throughout the experiment as 330 ± 3 °C. Following irradiation, specimens were lightly polished with colloidal silica in order to remove any surface implantation. The irradiated region was located by automated profile micro indentation testing, using a Struers Durascan automated indenter. A load of 0.005 kg was applied for 10 seconds; the use of a low load ensured indentations did not penetrate through the irradiated layer. Due to a slight aperture misalignment, the irradiated region over the sample set had a trapezoidal area. The area was measured and used to recalculate the nominal damage using the accumulated charge during irradiation. An average increase in hardness of 36 HV was recorded in the irradiated region relative to the non-irradiated region, as shown in Fig. 7.

### Method 1: XRD and DIC monitoring of plastic deformation

**Figure 8:**
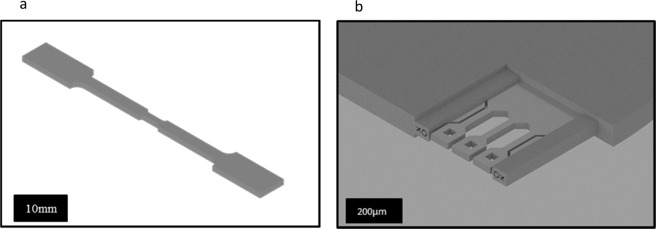
Specimen geometries for the two types of tensile samples (**a**) XRD-DIC sample, with a modified sub-gauge section of 5 × 1 × 1 mm^3 ^^74^, and (**b**) PFIB – micro-tensile sample, with a gauge section of ~150 × 30 × 30 µm^3^ ^36,74^.

As the irradiated region in the gauge section was discontinuous, the specimen was further modified by EDM, as shown in Fig. 8(a). The “double dog bone” geometry was designed so that the widest point of each radius leading into the second parallel section intersected with the start and end of the irradiated region. The inner gauge section was 4 mm ×1 mm ×1 mm, with a 0.5 mm radius in the transition region. Removal of the material ensured the experiment remained uniaxial whilst also removing the indentations used to locate the irradiated region. Samples were painted with a white anti-reflective coating and dusted over with black spray paint to apply a speckle pattern enabling the utilisation of digital image correlation (DIC) for monitoring strain.

The *in-situ* X-ray stress analysis and image collection strain analysis was carried out according to the methodology outlined in^35^. Quasistatic tensile loading was applied using a Kammrath and Weiss 5 kN tension-compression microtester at a displacement rate of 5 μm s^−1^, which corresponds to a strain rate of ~1.25 × 10^−3^ s^−1^. The crosshead was stopped at numerous hold points throughout the test to allow for image acquisition and X-ray diffraction measurements. Optical images were analysed using the commercially available LaVision DaVis 8.1.5 image correlation software, with the total strain averaged over the second gauge section.

Single peak X-ray stress analysis was performed on a Proto portable iXRD system using the sin^2^Ѱ technique on the {211} reflection using Cr Kα radiation giving a 2θ of 155°. Side inclination measurements were taken at 11 ψ-tilt angles between ±25° with 10 exposures using a 1 mm circular aperture. Due to the reduced aperture, the counting times were increased to 3 seconds for each of the exposures and ±2° goniometer undulation in χ was applied to increase counting statistics. Peak position and shape was determined using a gaussian fit, with stress calculated as follows:

6 $${\sigma }_{x}={\left(\frac{E}{1+v}\right)}_{hkl}\frac{\delta {d}_{x\psi }^{hkl}}{\delta si{n}^{2}\psi }\frac{1}{{d}_{\psi =0}}$$

where ${d}_{x\psi }^{hkl}$ is the inclined lattice spacing rotated around an axis normal to the loading direction, d_ψ=0_ is the stress-free lattice spacing at ψ = 0° and ${\left(\frac{E}{1+v}\right)}_{hkl}$ are the effective elastic constants of the diffracting plane (1/2 S2) calculated to be 5.94 × 10^−6^ MPa^−1^ ^35^.

The diffraction elastic constants were obtained, using the methodology outlined in ASTM E1426 -98^38^, which was to record Sin^2^ ψ plots for the {211} planes at increasing/decreasing load, through a number of repeating cycles. The gradients of the Sin^2^ ψ are plotted against stress allowing for the calculation of the diffraction elastic constants for this grain family by the gradient of the plot. An underpinning requirement for the measurement of applied plastic stresses using x-ray diffraction is that the interplanar response to applied stress remains linear outside of the elastic regime. A deviation in linearity during plastic deformation would cause an over or under representation of the applied stress. Selection of the correct planes for stress measurement is largely empirical but relates to the response to intergranular strains during deformation. The plane with the most linear response, with high enough intensity sitting within the measurement range of the goniometer is selected for stress measurement, conventionally it is the 211 plane family for BCC materials and the 311 family for FCC materials^72^.

Due to the low dose tested in this experiment, the diffraction elastic constants were assumed to be unchanged by irradiation since it is likely that point defects and dislocation loops would be present at these low doses and therefore would not significantly alter the interplanar response. In the standard case of residual stress measurement in plastically deformed surfaces using the Sin^2^ ψ method, such as those achieved using tooling or shot peening, the presence of dislocations does not alter the diffraction elastic constants^73^, so can be thought of as insensitive to this type of defect. However, in the case of materials irradiated to high doses, such as those leading to the formation of voids, precipitation and significant microstructural modification, the diffraction elastic constants would need to be calculated for this condition.

### Method 2: Plasma Focussed Ion Beam (PFIB) Milling and Piezo-mechanical testing

A coupon was prepared for PFIB milling by grinding and polishing the non-irradiated face of the EDM sample. It was mounted with the irradiated face down and carefully thinned by removal of the non-irradiated side using successively finer grades of silicon carbide paper (600, 800, 1200, 2500 and 4000 grit) until a thickness of ~60 μm was achieved. The foil was then carefully polished using diamond paste, starting with 3 μm and finishing with 0.25 μm, a final polishing step was carried out using colloidal silica to ensure a mirror finish. Subsequent FIB milling is expected to have removed any surface deformation introduced by previous mechanical polishing steps. After polishing, a 3 mm disk was extracted from the foil using a standard transmission electron microscope specimen punch, followed by grinding with fine grit silicon carbide paper to remove burrs. The foil was mounted between two glass slides with wax and ground to apply a straight edge. Attachment of the disk to a specimen mount using cyanoacrylate adhesive readied the specimen for preparation by PFIB. Specimens were prepared using the methodology outlined in^36^, with all milling performed at 30 kV and 2° overtilt using a PFIB equipped FEI Helios FEG-SEM. The high current (1.3 µA) ion milling was carried out from the non-irradiated side to preserve as much irradiated material as possible. This high current thinning step continued until a thickness of ~40 µm was reached. A subsequent automated cross polishing routine was performed using FEI Auto Slice and View 4 software package at 180 nA, removing approximately 5 µm from each side. Therefore, the through thickness dimension contained only the flat portion of the dose profile with the Bragg peak removed (Fig. 6c)). A final lamella width of 400 µm with a 30 µm thickness provided sufficient area to prepare three parallel tensile specimens (Fig. 8b). The final specimen geometry was milled at a beam current of 180 nA and the final gauge section dimensions were 150 µm × 30 µm × 30 µm, comprising of ~1600 laths given an approximate lath size of ~9 × 3 × 3 µm^3^.

Two specimens were tested to failure using a Microtesting Solutions (Hilliard, OH, USA) µ-Test Rig (MTR-3), mounted in a Zeiss Sigma FEG-SEM. Loading conditions were the same as those applied in ref. ^36^, with specimens loaded using a diamond pin at a rate of ~100 nm s^−1^ and hold points at 200 nm increments. Secondary electron images were taken at each hold point to provide an accurate method of strain measurement. Strain was calculated by tracking platinum fiducial markers using digital image correlation, as outlined in^36^.
